# Ultrasonographic Features of Papillary Thyroid Carcinomas According to Their Subtypes

**DOI:** 10.3389/fendo.2018.00223

**Published:** 2018-05-08

**Authors:** Hye Jin Baek, Dong Wook Kim, Gi Won Shin, Young Jin Heo, Jin Wook Baek, Yoo Jin Lee, Young Jun Cho, Ha Kyoung Park, Tae Kwun Ha, Do Hun Kim, Soo Jin Jung, Ji Sun Park, Ki Jung Ahn

**Affiliations:** ^1^Department of Radiology, Gyeongsang National University School of Medicine, Gyeongsang National University Hospital, Changwon, South Korea; ^2^Department of Radiology, Busan Paik Hospital, Inje University College of Medicine, Busan, South Korea; ^3^Department of General Surgery, Busan Paik Hospital, Inje University College of Medicine, Busan, South Korea; ^4^Department of Otorhinolaryngology-Head and Neck Surgery, Busan Paik Hospital, Inje University College of Medicine, Busan, South Korea; ^5^Department of Pathology, Busan Paik Hospital, Inje University College of Medicine, Busan, South Korea; ^6^Department of Nuclear Medicine, Busan Paik Hospital, Inje University College of Medicine, Busan, South Korea; ^7^Department of Radiation Oncology, Busan Paik Hospital, Inje University College of Medicine, Busan, South Korea

**Keywords:** thyroid nodule, papillary thyroid carcinoma, malignancy, subtype, ultrasonography

## Abstract

**Background:**

The ultrasonographic characteristics and difference for various subtypes of papillary thyroid carcinoma (PTC) are still unclear. The aim of this study was to compare the ultrasonographic features of PTC according to its subtype in patients undergoing thyroid surgery.

**Methods:**

In total, 140 patients who underwent preoperative thyroid ultrasonography (US) and thyroid surgery between January 2016 and December 2016 were included. The ultrasonographic features and the Korean Thyroid Imaging Reporting and Data System (K-TIRADS) category of each thyroid nodule were retrospectively evaluated by a single radiologist, and differences in ultrasonographic features according to the PTC subtype were assessed.

**Results:**

According to histopathological analyses, there were 97 classic PTCs (62.2%), 34 follicular variants (21.8%), 5 tall cell variants (3.2%), 2 oncocytic variants (1.3%), 1 Warthin-like variant (0.6%), and 1 diffuse sclerosing variant (0.6%). Most PTCs were classified under K-TIRADS category 5. Among the ultrasonographic features, the nodule margin and the presence of calcification were significantly different among the PTC subtypes. A spiculated/microlobulated margin was the most common type of margin, regardless of the PTC subtype. In particular, all tall cell variants exhibited a spiculated/microlobulated margin. The classic PTC group exhibited the highest prevalence of intranodular calcification, with microcalcification being the most common. The prevalence of multiplicity and nodal metastasis was high in the tall cell variant group.

**Conclusion:**

The majority of PTCs in the present study belonged to K-TIRADS category 5, regardless of the subtype. Our findings suggest that ultrasonographic features are not useful for distinguishing PTC subtypes.

## Introduction

Papillary thyroid carcinoma (PTC) is the most common type of thyroid malignancy, with an indolent clinical course and a favorable prognosis (1, 2). Several PTC subtypes exhibiting a combination of specific growth patterns, cell types, stromal changes, and nuclear features have been documented (3). In the revised American Thyroid Association guidelines (4), PTC is classified into three major subtypes according to the biological behavior: subtypes associated with aggressive outcomes, including the tall cell, columnar cell, and hobnail variants; subtype associated with less favorable outcomes, including the solid and diffuse sclerosing variants; and subtype associated with favorable outcomes, including the follicular, cribriform–morular, and Warthin-like variants. However, there is little information about the ultrasonographic features of PTC subtypes, even though ultrasonography (US) is routinely used as the primary imaging modality for the evaluation of thyroid nodules in daily clinical practice.

Recently, two review articles provided a brief summary about the ultrasonographic features of PTC subtypes that may be helpful for predicting the biological behavior and facilitating individualized management (3, 5). However, the ultrasonographic characteristics and difference of various PTC subtypes are still unclear. Therefore, the purpose of the present study was to investigate the ultrasonographic features of PTC according to its subtype in patients undergoing thyroid surgery.

## Materials and Methods

### Study Population

This retrospective study was approved by the appropriate institutional review board (IRB 17-0213), and the need for informed consent was waived. In total, 156 patients (138 women and 18 men; mean age, 51.9 ± 10.8 years; range, 16–79 years) underwent thyroid surgery for PTC between January 2016 and December 2016. From these, 16 patients were excluded because of the lack of preoperative US data (*n* = 7), presence of small nodules measuring <5 mm (*n* = 8), and poor US image quality (*n* = 1). Eventually, 140 PTCs (mean diameter, 10.6 ± 5.9 mm; range, 5–32.5 mm) in 140 patients (124 women and 16 men; mean age, 52.1 ± 11.1 years; range, 16–79 years) were included in our study.

### Thyroid US

Two radiologists (5 and 15 years of experience in performing thyroid US) performed all US examinations using one of two high-resolution US systems: iU 22 (Philips Medical Systems, Bothell, WA, USA); or the Aplio SSA-770A (Toshiba Medical Systems, Tokyo, Japan). A 5–12 or an 8–15-MHz linear-array transducer was used. During color Doppler US, a low pulse repetition frequency (700 Hz), low velocity scale (4.0 or 5.0 cm/s), and gain setting (between 75 and 78) were routinely used for evaluating thyroid nodules and parenchymal vascularity. The color Doppler gain was controlled such that perithyroidal fatty tissue did not display any random color noise.

### Image Analysis

In July 2017, a single radiologist (15 years of experience in performing thyroid US) retrospectively investigated all the ultrasonographic features of the 140 PTCs using a picture archiving and communication system. This radiologist was blinded to the PTC subtypes. The assessed features included the composition, echogenicity, margin, calcification status, shape, orientation, and vascularity (6, 7). According to the composition, nodules were classified as solid (no obvious cystic component), predominantly solid (solid component accounting for ≥50% of the total volume), predominantly cystic (cystic component accounting for >50% of the total volume), or cystic (no solid component). According to the echogenicity, nodules were classified as isoechoic (echogenicity same as that of the adjacent thyroid parenchyma), hypoechoic (decreased echogenicity compared with that of the adjacent thyroid parenchyma), and hyperechoic (increased echogenicity compared with that of the adjacent thyroid parenchyma). The nodule margin was classified as smooth, spiculated/microlobulated, or poorly defined, while the calcification status was classified as no calcification, presence of microcalcifications (echogenic foci measuring ≤1 mm with or without posterior acoustic shadowing), presence of macrocalcifications (echogenic foci measuring >1 mm with posterior shadowing), and presence of mixed calcification (micro- and macrocalcification). The nodule shape was classified as ovoid-to-round or irregular. The orientation of the nodule was classified as parallel (anteroposterior diameter equal to or less than the transverse or longitudinal diameter in the transverse or longitudinal plane) or non-parallel (anteroposterior diameter greater than the transverse or longitudinal diameter in the transverse or longitudinal plane). The degree of vascularity was classified as iso (vascularity same as that of the adjacent thyroid parenchyma), decreased (decreased vascularity compared with that of the adjacent thyroid parenchyma), or increased (increased vascularity compared with that of the adjacent thyroid parenchyma), while the pattern of vascularity was classified as central, peripheral, or mixed (central and peripheral).

On the basis of a retrospective analysis of the ultrasonographic features, each thyroid nodule was classified into Korean Thyroid Imaging Reporting and Data System (K-TIRADS) categories 3–5 (7). Suspicious ultrasonographic features included microcalcification, a spiculated/microlobulated margin, and a non-parallel orientation. Isoechoic or hyperechoic solid thyroid nodules without suspicious features were classified under K-TIRADS category 3 (low suspicion). Hypoechoic solid thyroid nodules with no suspicious features were classified under K-TIRADS category 4 (intermediate suspicion). Finally, hypoechoic solid thyroid nodules with any of the three suspicious features were classified under K-TIRADS category 5 (high suspicion).

### Histopathological Analysis

Histopathological analysis for determining the PTC subtype was retrospectively performed by a single pathologist with special expertise in thyroid tumors. All histopathological slides were reviewed according to the criteria of the World Health Organization International Classification of Thyroid Tumors (8). A tumor with conventional papillary features and completely surrounded by a fibrous capsule was classified as the encapsulated variant. A tumor exhibiting an exclusive follicular growth pattern was classified as the follicular variant, which was further stratified into infiltrative and encapsulated types. Encapsulated focal and minimally invasive lesions were considered encapsulated follicular variants. When the height was two or three times the width for >30% tumor cells, it was classified as the tall cell variant. The Warthin-like variant was diagnosed when histopathological features similar to those of Warthin’s tumors, which involve the salivary glands, were observed. The oncocytic variant was diagnosed when a papillary tumor was entirely composed of oncocytic cells. The diffuse sclerosing variant was a multifocal lobulated lesion characterized by the diffuse involvement of at least one thyroid lobe, fibrous stroma, dense lymphocytic infiltration, and abundant psammoma bodies.

### Statistical Analysis

Differences in ultrasonographic features according to the histopathological subtype of PTC were evaluated using Pearson’s chi-square test or, for small values, Fisher’s exact test for categorical variables. We excluded the Warthin-like and diffuse sclerosing variants from the statistical comparison of individual ultrasonographic features because there was only one case. All statistical analyses were performed using statistical software (SPSS, version 24.0, SPSS, Chicago, IL, USA). A *P*-value of <0.05 was considered statistically significant.

## Results

In total, 64 (45.7%) and 76 (54.3%) patients underwent hemithyroidectomy and total thyroidectomy, respectively. The histopathological diagnoses of the resected PTCs were as follows: classic PTC (97/140, 69.3%), follicular variant (34/140, 24.3%), tall cell variant (5/140, 3.6%), oncocytic variant (2/140, 1.4%), Warthin-like variant (1/140, 0.7%), and diffuse sclerosing variant (1/140, 0.7%). All 140 PTCs revealed a solid composition on US.

Multiplicity was observed in 51 lesions (36.4%), including 36 classic PTCs (37.1%), 10 follicular variants (29.4%), 4 tall cell variants (80%), and 1 oncocytic variant (50%); the Warthin-like variant did not exhibit multiplicity. There was no significant difference in the prevalence of multiplicity among the PTC subtypes (*P* = 0.231). Nodal metastasis was identified in association with 54 lesions (38.6%), including 43 classic PTCs (44.3%), 5 follicular variants (14.7%), 4 tall cell variants (80%), 1 Warthin-like variant (100%), and 1 diffuse sclerosing variant (100%); a significant difference was noted among the various subtypes (*P* < 0.0001).

**Figure 1:**
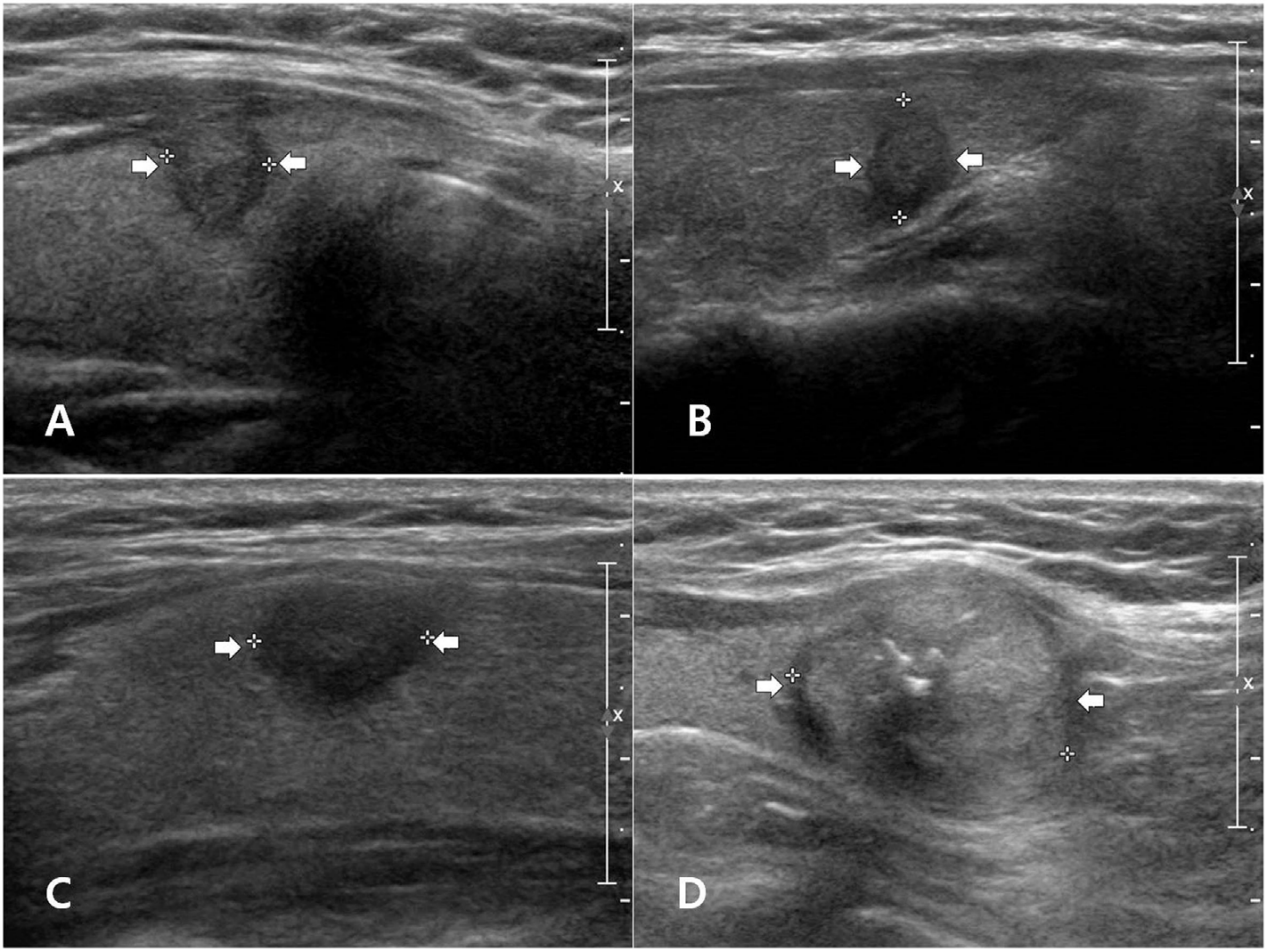
Examples of papillary thyroid carcinoma (PTC) subtypes with malignant ultrasonographic feature(s) on longitudinal gray-scale sonograms: classic PTC **(A)**, follicular variant **(B)**, tall cell variant **(C)**, and oncocytic variant **(D)**.

The ultrasonographic features of the various PTC subtypes are summarized in Table 1. The common sonographic features of PTCs included hypoechogenicity, spiculated/microlobulated margin, none- or microcalcification, ovoid-to-round shape, non-parallel orientation, and iso-degree and mixed pattern of vascularity, regardless of the PTC subtype. There were no differences among variants with regard to most of the ultrasonographic features (Figure 1). Only two features, namely the margin and calcification status, were significantly different among subtypes. A spiculated/microlobulated margin was the most common type of margin, regardless of the PTC subtype. In particular, all tall cell variants exhibited a spiculated/microlobulated margin. The classic PTC group exhibited the highest prevalence of intranodular calcification, regardless of the type, with microcalcification being the most common. By contrast, the follicular variants appeared as solid nodules without calcification, while the tall cell and oncocytic variants did not exhibit microcalcification. Other ultrasonographic features, including echogenicity, shape, orientation, degree of vascularity, pattern of vascularity, and K-TIRADS category, were comparable among subtypes. Most PTCs exhibited a non-parallel orientation and were classified under K-TIRADS category 5, regardless of the subtype. In particular, all tall cell and oncocytic variants showed a non-parallel orientation and were classified under K-TIRADS category 5.

**Table 1:** Comparison of ultrasonographic features of PTC according to the subtype.

Subtype US features	Classic (*n* = 97)	Follicular variant (*n* = 34)	Tall cell variant (*n* = 5)	Oncocytic variant (*n* = 2)	*P* value
Echogenicity				0.483
Iso-	11 (11.3)	5 (14.7)	0	1 (50)	
Hypo-	84 (86.6)	29 (85.3)	5 (100)	1 (50)	
Hyper-	2 (2.1)	0	0	0	
Margin					0.023
Smooth	14 (14.4)	12 (35.3)	0	1 (50)	
Spiculated/microlobulated	83 (85.6)	22 (64.7)	5 (100)	1 (50)	
Poorly defined	0	0	0	0	
Calcification					0.005
None	44 (45.4)	21 (61.8)	4 (81)	1 (50)	
Micro-	40 (41.2)	6 (17.6)	0	0	
Macro-	2 (2.1)	4 (11.8)	1 (20)	1 (50)	
Mixed	11 (11.3)	3 (8.8)	0	0	
Shape					0.631
Ovoid-to-round	94 (96.9)	34 (100)	5 (100)	2 (100)	
Irregular	3 (3.1)	0	0	0	
Orientation					0.872
Non-parallel	88 (90.7)	30 (88.2)	5 (100)	2 (100)	
Parallel	9 (9.3)	4 (11.8)	0	0	
Degree of vascularity					0.551
Iso-	75 (77.3)	25 (73.5)	4 (80)	1 (50)	
Decreased	0	1 (2.9)	0	0	
Increased	20 (20.6)	7 (20.6)	1 (20)	1 (50)	
Pattern of vascularity					0.348
Peripheral	8 (8.2)	8 (23.5)	0	0	
Central	2 (2.1)	0	0	0	
Mixed	85 (87.6)	25 (73.5)	5 (100)	2 (100)	
K-TIRADS category					0.290
3	0	2 (5.9)	0	0	
4	4 (4.1)	1 (2.9)	0	0	
5	93 (95.9)	31 (91.2)	5 (100)	2 (100)	

*Data presented in parentheses are percentage of each item*.

*US, ultrasonography; PTC, papillary thyroid carcinoma; K-TIRADS, Korean Thyroid Imaging Reporting and Data System*.

*Of the 138 cases, nodule vascularity was analyzed in 135 cases*.

The 34 follicular variants included 30 infiltrative (88.2%) and 4 encapsulated (11.8%) lesions. The ultrasonographic features of the follicular variants according to the two subgroups are listed in Table 2. No significant difference was observed in any feature between the two subgroups.

**Table 2:** Ultrasonographic features of encapsulated and infiltrative follicular variants of PTC.

Subtype US features	Encapsulated (*n* = 4)	Infiltrative (*n* = 30)	*P* value
Echogenicity			1.000
Iso-	0	5 (16.7)	
Hypo-	4 (100)	25 (83.3)	
Hyper-	0	0	
Margin			0.115
Smooth	3 (75)	9 (30)	
Spiculated/microlobulated	1 (25)	21 (70)	
Poorly defined	0	0	
Calcification			0.699
None	3 (75)	18 (60)	
Micro-	0	6 (20)	
Macro-	1 (25)	3 (10)	
Mixed	0	3 (10)	
Shape			NA
Ovoid-to-round	4 (100)	30 (100)	
Irregular	0	0	
Orientation			0.409
Non-parallel	3 (75)	27 (90)	
Parallel	1 (25)	3 (10)	
Degree of vascularity			0.145
Iso-	2 (50)	23 (76.7)	
Decreased	1 (25)	0	
Increased	1 (25)	6 (20)	
Pattern of vascularity			1.000
Peripheral	1 (25)	7 (23.3)	
Central	0	0	
Mixed	3 (75)	22 (73.3)	
K-TIRADS category			1.000
3	0	2 (6.7)	
4	0	1 (3.3)	
5	4 (100)	27 (90)	

*Data presented in parentheses are percentage of each item*.

*NA, not applicable; US, ultrasonography; PTC, papillary thyroid carcinoma; K-TIRADS, Korean Thyroid Imaging Reporting and Data System*.

*Of the 34 cases, nodule vascularity was analyzed in 33 cases*.

## Discussion

Papillary thyroid carcinoma is known to exhibit an indolent clinical course and a favorable prognosis (1, 2). However, recent studies and revised American Thyroid Association guidelines have reported that different histopathological subtypes of PTCs exhibit different clinical courses and prognoses, and that the ultrasonographic characteristics may be helpful for predicting the subtype (3–5, 9–11). To our knowledge, no study has objectively compared the ultrasonographic features of different PTC subtypes.

In the present study, the majority of PTCs were classified under K-TIRADS category 5, and the tall cell variant showed an aggressive behavior with a high prevalence of multiplicity and nodal metastasis. The ultrasonographic features identified in the present study were similar to those reported in two previous studies of PTC subtypes (3, 5). These studies reported that the tall cell variant typically exhibits malignant features with frequent nodal metastasis (3, 5). However, they did not report specific features for each PTC subtype because of a high proportion of classic PTCs and wide overlap of ultrasonographic features among subtypes.

The follicular variant of PTC tends to appear benign on US and is more similar to follicular neoplasms than to PTCs (3, 5, 9, 10). However, no previous studies have compared ultrasonographic features between infiltrative and encapsulated follicular variants. In the present study, most follicular variants exhibited highly suspicious features on US, and all four encapsulated types were classified under K-TIRADS category 5. The reason for this difference is unclear. Furthermore, there was no significant difference in any ultrasonographic feature between the infiltrative and encapsulated types. In addition, most of the follicular variants did not exhibit calcification. However, only four encapsulated follicular variants were included in our study. For more clarity, further studies assessing a greater number of specimens may be required.

According to previous studies, Warthin-like variants can be misdiagnosed as possibly benign nodules because they generally do not exhibit suspicious ultrasonographic features such as a spiculated/microlobulated margin, a non-parallel orientation, and the presence of microcalcification (3, 5, 11, 12). However, the Warthin-like variant in the present study exhibited two suspicious features on US and was classified under K-TIRADS category 5.

Several limitations of this study should be considered while interpreting the results. First, there was an unavoidable selection bias because the data for all patients were retrospectively evaluated. Second, all study patients underwent thyroid surgery. Although this factor was necessary for correlating ultrasonographic features with the histopathological findings as a reference standard, sampling bias may have occurred. Third, a relatively high proportion of classic PTCs was included because of the low incidence of other PTC subtypes; this could have affected our results. Finally, the sample size was small. In particular, several variants, including solid, columnar cell, hobnail, and cribriform–morular PTCs, were not included. Therefore, further studies with a larger sample size and more PTC subtypes are necessary to further clarify our findings.

In conclusion, the majority of PTCs were classified under K-TIRADS category 5 and exhibited overlapping suspicious ultrasonographic features. These findings suggest that ultrasonographic features are not useful for distinguishing the various subtypes of PTC.

## Ethics Statement

This study follows the principles expressed in the Declaration of Helsinki. All study participants waived informed consents owing to the retrospective analysis, and the study design was approved by the appropriate ethics review boards (IRB 17-0213).

## Author Contributions

Concept and design: DWK. Acquisition of data, literature review, and refinement of manuscript: All authors. Analysis and interpretation of data: HB and DWK. Manuscript writing: HB. Review of final manuscript: DWK.

## Conflict of Interest Statement

The authors declare that the research was conducted in the absence of any commercial or financial relationships that could be construed as a potential conflict of interest.

## References

[1] Nam SY Shin JH Han BK Ko EY Ko ES Hahn SY Preoperative ultrasonographic features of papillary thyroid carcinoma predict biological behavior. J Clin Endocrinol Metab (2013) 98:1476–82. 10.1210/jc.2012-4072 23463652

[2] Ito Y Miyauchi A Kihara M Takamura Y Kobayashi K Miya A. Relationship between prognosis of papillary thyroid carcinoma patient and age: a retrospective single-institution study. Endocr J (2012) 59:399–405. 10.1507/endocrj.EJ11-0288 22374240

[3] Shin JH. Ultrasonographic imaging of papillary thyroid carcinoma variants. Ultrasonography (2017) 36:103–10. 10.14366/usg.16048 28222584 PMC5381844

[4] Haugen BR Alexander EK Bible KC Doherty GM Mandel SJ Nikiforov YE American Thyroid Association management guidelines for adult patients with thyroid nodules and differentiated thyroid cancer: The American Thyroid Association guidelines task force on thyroid nodules and differentiated thyroid cancer. Thyroid (2016) 26:1–133. 10.1089/thy.2015.0020 26462967 PMC4739132

[5] Lee JH Shin JH Lee HW Oh YL Hahn SY Ko EY. Sonographic and cytopathologic correlation of papillary thyroid carcinoma variants. J Ultrasound Med (2015) 34:1–15. 10.7863/ultra.34.1.1 25542934

[6] Moon WJ Baek JH Jung SL Kim DW Kim EK Kim JY Ultrasonography and ultrasound-based management of thyroid nodules: consensus statement and recommendations. Korean J Radiol (2011) 12:1–14. 10.3348/kjr.2011.12.1.1 21228935 PMC3017873

[7] Shin JH Baek JH Chung J Ha EJ Kim JH Lee YH Ultrasonography diagnosis and imaging-based management of thyroid nodules: revised Korean society of thyroid radiology consensus statement and recommendations. Korean J Radiol (2016) 17:370–95. 10.3348/kjr.2016.17.3.370 27134526 PMC4842857

[8] Li Volsi VA Albores-Saavedra J Asa SL Baloch ZW Sobrinho-Simões M Wenig B Papillary carcinoma. 3rd ed In: DeLellis RA Lloyd RV Heitz PU Eng C, editors. Pathology and Genetics: Tumours of Endocrine Organs. Lyon/France: The International Agency of Research on Cancer Press/World Health Organization Classification of Tumours (2004). p. 57–66.

[9] Rhee SJ Hahn SY Ko ES Ryu JW Ko EY Shin JH. Follicular variant of papillary thyroid carcinoma: distinct biologic behavior based on ultrasonographic features. Thyroid (2014) 24:683–8. 10.1089/thy.2013.0351 24341498

[10] Choi JW Kim TH Roh HG Moon WJ Lee SH Hwang TS Radiologic and pathologic findings of a follicular variant of papillary thyroid cancer with extensive stromal fat: a case report. Korean J Radiol (2015) 16:1349–52. 10.3348/kjr.2015.16.6.1349 26576126 PMC4644758

[11] Kim GR Shin JH Hahn SY Ko EY Oh YL. Ultrasonographic features and clinical characteristics of Warthin-like variant of papillary thyroid carcinoma. Endocr J (2016) 63:329–35. 10.1507/endocrj.EJ15-0620 26806192

[12] Moon WJ Jung SL Lee JH Na DG Baek JH Lee YH Benign and malignant thyroid nodules: US differentiation-multicenter retrospective study. Radiology (2008) 247:762–70. 10.1148/radiol.2473070944 18403624

